# Dysphagia in primary progressive aphasia: Clinical predictors and neuroanatomical basis

**DOI:** 10.1111/ene.16370

**Published:** 2024-06-21

**Authors:** Salvatore Mazzeo, Eoin Mulroy, Jessica Jiang, Michael Lassi, Jeremy C. S. Johnson, Chris J. D. Hardy, Jonathan D. Rohrer, Jason D. Warren, Anna Volkmer

**Affiliations:** ^1^ Dementia Research Centre, Department of Neurodegenerative Disease, UCL Queen Square Institute of Neurology University College London London UK; ^2^ Research and Innovation Centre for Dementia–CRIDEM University of Florence, Azienda Ospedaliera–Universitaria Careggi Florence Italy; ^3^ Vita‐Salute San Raffaele University Milan Italy; ^4^ IRCCS Policlinico San Donato San Donato Milanese Italy; ^5^ BioRobotics Institute and Department of Excellence in Robotics and AI Scuola Superiore Sant'Anna Pisa Italy; ^6^ Department of Psychology & Language Sciences University College London London UK

**Keywords:** Alzheimer dementia, dysphagia, primary progressive aphasia, semantic dementia, swallowing

## Abstract

**Background and purpose:**

Dysphagia is an important feature of neurodegenerative diseases and potentially life‐threatening in primary progressive aphasia (PPA) but remains poorly characterized in these syndromes. We hypothesized that dysphagia would be more prevalent in nonfluent/agrammatic variant (nfv)PPA than other PPA syndromes, predicted by accompanying motor features, and associated with atrophy affecting regions implicated in swallowing control.

**Methods:**

In a retrospective case–control study at our tertiary referral centre, we recruited 56 patients with PPA (21 nfvPPA, 22 semantic variant [sv]PPA, 13 logopenic variant [lv]PPA). Using a pro forma based on caregiver surveys and clinical records, we documented dysphagia (present/absent) and associated, potentially predictive clinical, cognitive, and behavioural features. These were used to train a machine learning model. Patients' brain magnetic resonance imaging scans were assessed using voxel‐based morphometry and region‐of‐interest analyses comparing differential atrophy profiles associated with dysphagia presence/absence.

**Results:**

Dysphagia was significantly more prevalent in nfvPPA (43% vs. 5% svPPA and no lvPPA). The machine learning model revealed a hierarchy of features predicting dysphagia in the nfvPPA group, with excellent classification accuracy (90.5%, 95% confidence interval = 77.9–100); the strongest predictor was orofacial apraxia, followed by older age, parkinsonism, more severe behavioural disturbance, and more severe cognitive impairment. Significant grey matter atrophy correlates of dysphagia in nfvPPA were identified in left middle frontal, right superior frontal, and right supramarginal gyri and right caudate.

**Conclusions:**

Dysphagia is a common feature of nfvPPA, linked to underlying corticosubcortical network dysfunction. Clinicians should anticipate this symptom particularly in the context of other motor features and more severe disease.

## INTRODUCTION

Primary progressive aphasia (PPA) denotes a diverse group of neurodegenerative disorders led by insidious decline in speech and language [1]. There are three recognized canonical PPA syndromes [1, 2]: nonfluent/agrammatic variant (nfvPPA), associated with effortful, halting, and distorted (apraxic) speech and/or agrammatism, and a left anterior peri‐Sylvian atrophy profile; semantic variant (svPPA), associated with loss of vocabulary (impaired single word comprehension), and focal left anteromesial temporal lobe atrophy; and logopenic variant (lvPPA), associated with word‐finding difficulty and impaired phonological working memory, and left temporoparietal atrophy. It is increasingly recognized that these syndromes encompass a range of impairments beyond the domain of language, particularly later in their course [3, 4]. Dysphagia is potentially one of the most clinically significant issues in PPA; it is associated with increased risk of aspiration pneumonia, a leading cause of death in people with dementia [5], predicts shortened survival if it develops early [6], and constitutes an important milestone of later stage illness evolution [3, 4]. However, dysphagia in PPA syndromes has not been well characterized, and diagnosis and management of the issues it presents are often delayed [7].

Here, we set out to assess diagnostic, phenotypic, and neuroanatomical associations of dysphagia in a historical cohort of patients representing all major syndromes of PPA. We collected information about dysphagia prevalence and clinical predictors from structured assessments involving patients' caregivers and clinical records, and we applied a machine learning algorithm to evaluate the relative importance of dysphagia predictors. We assessed neuroanatomical correlates of dysphagia using voxel‐based morphometry (VBM) of patients' brain magnetic resonance imaging (MRI) scans. Based on available evidence in PPA and other neurodegenerative diseases [6, 8, 9, 10], we hypothesized that dysphagia would be more prevalent and develop earlier in patients with nfvPPA versus other PPA syndromes and would be associated with more severe motor dysfunction and behavioural disturbance. We further hypothesized that dysphagia in PPA would be associated with regional brain atrophy involving frontosubcortical structures previously implicated in control of swallowing in the healthy brain [11, 12] and in dysphagia accompanying stroke and parkinsonian disorders [13, 14, 15].

## MATERIALS AND METHODS

### Patient cohort and assessment of dysphagia

We retrospectively assessed all patients in our historical research cohort at the Dementia Research Centre who fulfilled consensus diagnostic criteria for a canonical PPA syndrome [1] and had relevant information about their swallowing function recorded. The cohort comprised 56 consecutive patients (21 [38%] nfvPPA, 22 [39%] svPPA, 13 [23%] lvPPA) recruited between 2016 and 2020. All had syndromes of mild to moderate severity and supportive brain MRI with minimal cerebrovascular burden. Patient group clinical characteristics are summarized in Table 1. Using a structured clinical survey, we recorded whether dysphagia had developed at the time of the initial research assessment ("dysphagia at baseline"), in consultation with each patient's primary caregiver or equivalent close informant.

**TABLE 1:** Clinical and behavioural characteristics of the primary progressive aphasia cohort.

Characteristic	nfvPPA			svPPA	lvPPA
	Combined	Dysphagia at baseline	No dysphagia at baseline		
*n* (% female)	21 (38)	9 (67)	12 (50)	22 (41)	13 (15)
Age at first assessment, years	71.6 (8.3)	74.3 (9.2)	69.5 (7.2)	66.2 (7.1)	67.5 (8.6)
Handedness, R/L	20/1	9/0	11/1	21/1	12/1
Formal education, years	13.8 (2.9)	14.1 (3.0)	13.6 (2.9)	14.2 (3.0)	14.9 (2.6)
Dysphagia at baseline, %	**43** ^a^, ^b^	100	0	**4.5** ^a^	**0** ^b^
Symptom duration, years	4.8 (2.6)	4.7 (1.8)	4.9 (3.4)	5.5 (2.5)^c^	4.7 (1.8)
MMSE	20.5 (9.5)	18.1 (11.1)	22.3 (8.1)	21.91 (7.8)	15.7 (8.7)
Primary progressive apraxia of speech, %^d^	9.5	11.1	8.3	NA	NA
Orofacial apraxia, %	**68.8** ^e^	**100** ^f^	**50.0** ^f^	**0** ^e^	38.5
Parkinsonism, %^g^	**57.1** ^h^, ^i^	**88.9** ^j^	**33.3** ^j^	**0** ^h^	**0** ^i^
Behavioural change					
CBI‐R total score	49.3 (30.9)	60.2 (38.0)	37.2 (14.7)	68.2 (33.9)	43.9 (29.6)
Table manners decline, median (IQR)	0.0 (0.0)	0.0 (3.0)	0.0 (0.0)	**0.5 (1.0)** ^k^	**0.0 (0.0)** ^k^
Increase in appetite, median (IQR)	0.0 (2.0)	0.0 (2.0)	0.0 (1.0)	0.0 (2.0)	0.0 (0.0)
Clinical follow‐up					
Interval, years	3.6 (1.6)	3.5 (1.3)	4.19 (1.55)	5.08 (1.4)	3.55 (1.1)
Dysphagia during follow‐up, %	NA	NA	**50** ^l^	**13.3** ^l^	28.6
Time to dysphagia during follow‐up, years	NA	NA	3.06 (1.0)	5.4 (1.3)	2.8 (0.9)

*Note*: The table presents mean (SD) values unless otherwise indicated. Bold indicates significant group differences; for all comparisons, the statistical significance criterion was *p* < 0.05.

Abbreviations: CBI‐R, Cambridge Behavioural Inventory–Revised; IQR, inter‐quartile range; L, left; lvPPA, patient group with logopenic variant primary progressive aphasia; MMSE, Mini‐Mental State Examination score; NA, not applicable; nfvPPA, patient group with nonfluent/agrammatic primary progressive aphasia; R, right; svPPA, patient group with semantic variant primary progressive aphasia.

^a^ χ^2^ = 9.81, *p* = 0.002, *V* = 0.483.

^b^ χ^2^ = 8.30, *p* = 0.004, *V* = 0.502.

^c^ The svPPA patient with dysphagia at baseline had illness duration of 6.3 years prior to assessment.

^d^ Only two cases in the nfvPPA group met criteria for "pure" primary progressive apraxia of speech, i.e., a clinical presentation dominated by speech apraxia with normal performance on linguistic tests (grammar comprehension and output, naming, single word comprehension when first assessed; see Table S1).

^e^ χ^2^ = 11.51, *p* = 0.001, *V* = 0.558.

^f^ χ^2^ = 4.36, *p* = 0.037, *V* = 0.552.

^g^ Two of 12 (16.7%) patients without dysphagia at baseline and six of nine (66.7%) patients with dysphagia at baseline had features of progressive supranuclear palsy/corticobasal syndrome.

^h^ χ^2^ = 8.89, *p* = 0.003, *V* = 0.667.

^i^ χ^2^ = 6.06, *p* = 0.014, *V* = 0.492, *Χ* ^2^ = 6.48.

^j^ *p* = 0.01, *V* = 0.556.

^k^ *W* = 4.15, *p* = 0.009.

^l^ χ^2^ = 9.81, *p* = 0.002, *V* = 0.546.

Patients underwent a comprehensive neuropsychological assessment (Table S1), and behavioural symptoms were assessed using the Cambridge Behavioural Inventory–Revised (CBI‐R) [16] (Table 1). In addition, patients had a neurological examination (by a neurologist with experience in movement disorders), noting the presence of orofacial apraxia, parkinsonism, and any features of corticobasal syndrome (CBS) or progressive supranuclear palsy (PSP). Orofacial apraxia was assessed by asking the patient to perform common orofacial gestures (licking lips, puffing out cheeks, blowing a kiss, protruding the tongue). Parkinsonism was recorded based on the presence of bradykinesia, rest tremor, rigidity, and/or a consistent abnormality of gait and posture. Features of CBS were recorded if at least one of the following (based on clinical diagnostic criteria) [17] was present: cortical sensory loss, alien limb phenomenon, limb akinesia, limb dystonia, or myoclonus. Features of PSP were recorded if at least one of the following (based on clinical diagnostic criteria) [18] was present: vertical supranuclear gaze palsy, impaired eyelid function, repeated unprovoked falls or tendency to fall on the pull‐test, or gait freezing.

For those patients with PPA in our research cohort without dysphagia at baseline who were also followed in the Cognitive Disorders Clinic at the National Hospital for Neurology and Neurosurgery, we assessed whether they subsequently developed dysphagia during their period of contact with the clinic ("dysphagia during follow‐up"). For all patients who developed dysphagia (either at baseline or during follow‐up), we assessed associated clinical features from their hospital records. Typically, patients attend clinic approximately 6‐monthly and are reviewed by a neurologist and (if any concerns about swallowing function are raised) a speech and language therapist. Using a structured pro forma, we extracted information about any food and fluid modifications, nutrition, aspiration events, and other important clinical consequences of dysphagia for activities of daily life. Based on this extracted information, dysphagia severity was scored (by A.V.) using the Functional Oral Intake Scale [19] and International Dysphagia Diet Standardisation Initiative [20]. Extracted clinical data are summarized in Table 2.

**TABLE 2:** Clinical assessment of swallowing function in patients with primary progressive aphasia.

Characteristic	Case 1^a^	Case 2^a^	Case 3^a^	Case 4	Case 5	Case 6	Case 7	Case 8	Case 9	Case 10
Diagnosis	nfvPPA	nfvPPA	nfvPPA	nfvPPA	nfvPPA	nfvPPA	nfvPPA	svPPA	svPPA	lvPPA
Dysphagia onset, years^b^	1	3	3	8	8	9	11	8	15	10
FOIS rating	6	4	5	5	NR	5	5	5	NR	NR
IDDSI: food	7	4	6	7	NR	6	6	6	NR	NR
IDDSI: fluid	0	0	0	0	NR	0	0	0	NR	NR
Compensatory strategies	Need for care	NR	Care when fatigued	Provides sensory feedback, food cut up	NR	Smaller portions, verbal/environmental prompts	Food cut up	NR	NR	NR
Chest infections^c^	NR	NR	NR	NR	NR	NR	NR	NR	6 months before swallow assessment	NR
Weight loss^c^	NR	NR	Gained weight	NR	NR	NR	Down 2 dress sizes	NR	NR	NR
Other concerns around swallowing	NR	Choking/coughing occasionally during meals	Eats slower, food on lips, clears throat during meals	Coughs occasionally during meals	Impulsive eating, coughing after eating	Cramming food	NR	Spitting food out, takes more time to swallow liquid	Coughing occasionally on drinking, vomited once	Protrusion of tongue when eating
Impact on daily life	NR	NR	Less able to attend family meals out	Needs support from carer for eating and drinking	NR	Partner anxious	NR	NR	NR	NR

*Note*: The information in the table was extracted by review of clinical records for those patients with primary progressive aphasia with dysphagia who were also followed up in the National Hospital for Neurology and Neurosurgery Cognitive Clinic (see text).

Abbreviations: FOIS, Functional Oral Intake Scale; IDDSI, International Dysphagia Diet Standardisation Initiative (fluid–level 0, thin; food–level 4, pureed; level 6, soft and bite‐sized; level 7, regular/easy to chew); lvPPA, logopenic variant primary progressive aphasia; nfvPPA, nonfluent/agrammatic variant primary progressive aphasia; NR, none reported; svPPA, semantic variant primary progressive aphasia.

^a^ Case 1, Case 2, and Case 3 had dysphagia at baseline (Cases 4–10 developed dysphagia during follow‐up).

^b^ Years following onset of illness.

^c^ Absence of this symptom was not recorded systematically.

The study was approved by the University College London institutional ethics committee (reference number: 06/Q0512/52). All participants gave informed consent in accordance with the Declaration of Helsinki.

### Analysis of clinical and neuropsychological data

All statistical analyses were performed using SPSS Statistics Software v25 (IBM, Armonk, NY, USA) and the computing environments R4.2.3 (R Foundation for Statistical Computing, Vienna, Austria; 2013) and Python v3.11.4 (Python Software Foundation, www.python.org). Normality of data distributions was assessed using the Shapiro–Wilk test. We conducted descriptive statistical tests to examine central tendency and variability of clinical data using means and SDs for continuous variables and percentages and 95% confidence intervals (CIs) for categorical variables, respectively. For normally distributed variables, we used *t*‐tests to compare groups pairwise and one‐way analysis of variance (ANOVA) with Bonferroni post hoc tests to compare more than two groups. For nonnormally distributed variables, we used Kruskal–Wallis tests with Dwass–Steel–Critchlow–Fligner pairwise comparisons. For categorical variables, we used χ^2^ tests to compare groups. We calculated effect sizes using Cohen's *d* for normally distributed data, *η* ^2^ for ANOVA, *ε* ^2^ for Kruskal–Wallis tests, and Cramer's *V* for categorical data.

To assess the relative importance of potentially relevant clinical characteristics in predicting dysphagia at baseline, candidate predictive variables identified from past clinical experience and/or the structured survey (presence of orofacial apraxia or parkinsonism, symptom duration, Mini‐Mental State Examination [MMSE] score, behavioural disturbances as indexed by total CBI‐R score, table manners decline or increased appetite) and general demographic characteristics (age at baseline, sex, handedness) were used as input features to train a random forest classifier. We applied this classifier only to the nfvPPA group (as there was just a single case of dysphagia at baseline in the rest of the cohort). We considered "dysphagic" and "nondysphagic" as output labels. For each feature, average importance was calculated across all cross‐validation folds, and features were ranked according to their importance. Feature importance values were compared by ANOVA with Bonferroni post hoc tests. Details of the machine learning model are provided in Appendix S1.

In addition, a Kaplan–Meyer (pairwise log‐rank comparisons) analysis was used to compare proportions of patients in each PPA syndromic group (across the whole cohort) who developed dysphagia during follow‐up. To evaluate the effect of clinical features on the development of dysphagia during follow‐up, we performed a backward multivariate Cox proportional hazard regression analysis considering follow‐up interval as time.

### Brain image acquisition and analysis

Volumetric T1‐weighted brain MRI scans acquired on a 3‐T MAGNETOM Prisma scanner (Siemens Healthineers, Erlangen, Germany) for 17 patients with nfvPPA (nine with dysphagia at baseline, eight without dysphagia either at baseline or during follow‐up) were entered into the VBM analysis. Image acquisition and preprocessing steps followed standard local protocols (details in Appendix S1). Voxel intensity (grey matter volume) was modelled as a function of presence versus absence of dysphagia at baseline. Patient age, total intracranial volume, and symptom duration (as a measure of disease severity) were included as covariates of no interest in the model. Patient subgroups with and without dysphagia at baseline were compared in two‐way contrasts at two different thresholds. For the purpose of reporting significant grey matter associations, we compared subgroups at a stringent significance threshold of *p* < 0.05 corrected for multiple voxelwise comparisons within neuroanatomical regions of interest (as prespecified in our prior hypotheses) [15, 21]. These regions comprised bilateral frontal poles, superior, middle and inferior frontal gyri, precentral gyri, supramarginal gyri, and basal ganglia, customized from the Oxford/Harvard brain maps in FSLview v3.1 [22, 23] to fit the group mean template brain image; the brain regions assessed are shown in Figure S1. In addition, to allow us to inspect the extent of any additional, weaker regional grey matter associations of dysphagia, we compared subgroups at a lenient significance threshold of *p* < 0.001 uncorrected for multiple voxelwise comparisons over the whole brain.

## RESULTS

### Characteristics of the PPA cohort and comparisons between syndromic groups

Clinical and behavioural characteristics of the PPA syndromic groups and results of between‐group comparisons are presented in Table 1; neuropsychological profiles aligned with syndromic diagnoses (Table S1). Of the 56 patients with PPA included in the research cohort, 10 had dysphagia at baseline; 30 patients (eight nfvPPA, 15 svPPA, seven lvPPA) were followed up clinically, and a further eight of these developed dysphagia during follow‐up (Figure S2). Detailed clinical record information was available for three patients who had dysphagia at baseline and seven who developed dysphagia during follow‐up (Table 2).

PPA syndromic groups did not differ significantly in age, sex, symptom duration, years of education, MMSE score, CBI‐R total score, or eating behaviours. Dysphagia at baseline was significantly more frequent in the nfvPPA group (nine cases, 43%) than in the svPPA group (one case, 5%; χ^2^ = 9.81, *p* = 0.002, *V* = 0.483) and the lvPPA group (no cases; χ^2^ = 8.30, *p* = 0.004, *V* = 0.502).

The nfvPPA group had a significantly higher prevalence of parkinsonism than both other PPA groups (svPPA, χ^2^ = 8.89, *p* = 0.003, *V* = 0.667; lvPPA, χ^2^ = 6.06, *p* = 0.014, *V* = 0.492), including eight cases with features of PSP or CBS, and a significantly higher prevalence of orofacial apraxia than the svPPA group (χ^2^ = 11.51, *p* = 0.001, *V* = 0.558). PPA groups differed significantly in prevalence of decline in table manners (*p* = 0.009, *ε* ^2^ = 0.20), which in a post hoc pairwise analysis was significantly more prevalent in the svPPA group than the lvPPA group (Wald (W) = 4.15, *p* = 0.009).

### Factors predicting dysphagia at baseline (in nfvPPA)

**FIGURE 1:**
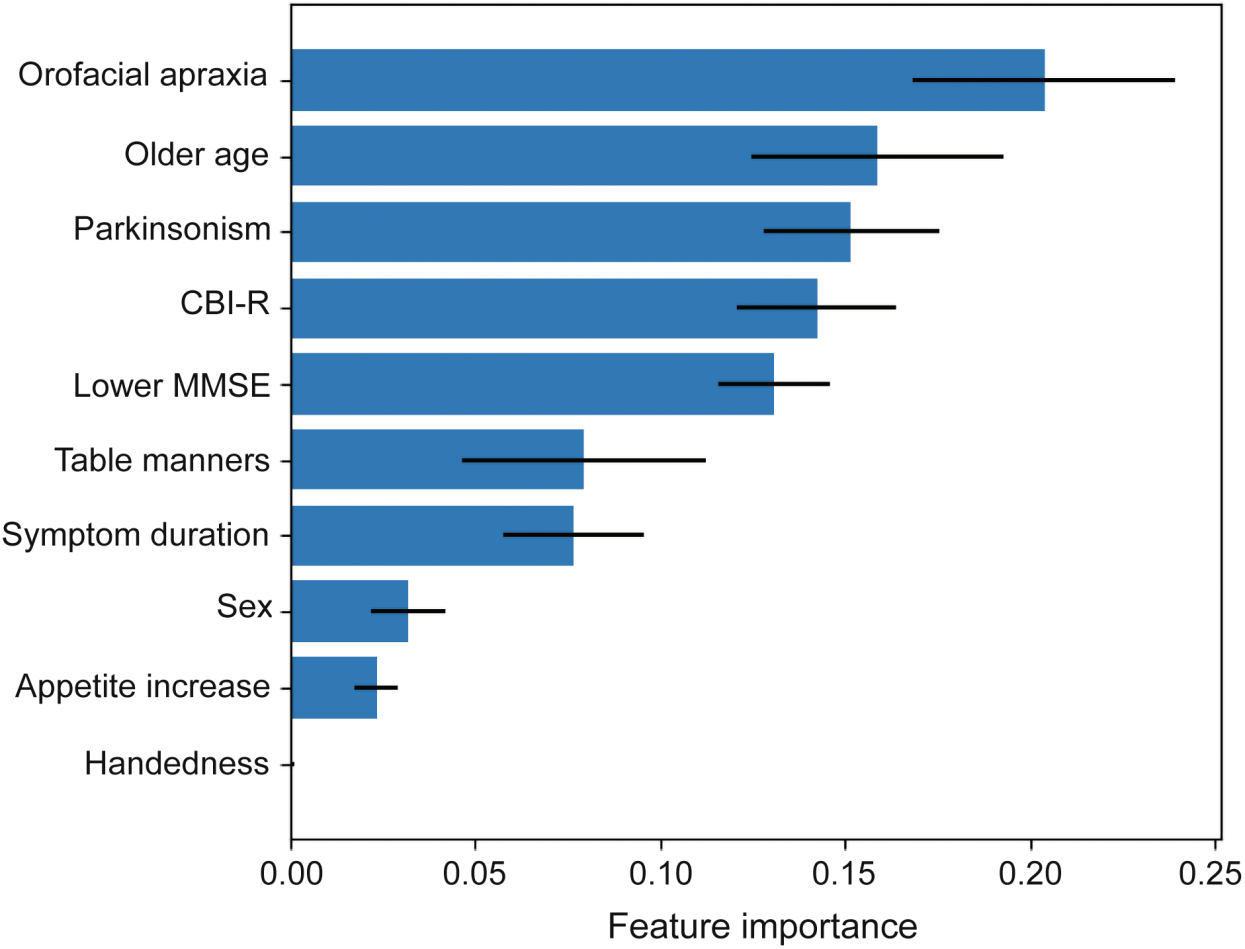
Bar chart showing the relative importance (with 95% confidence intervals) of clinical features associated with dysphagia (at baseline) in patients with nonfluent/agrammatic variant primary progressive aphasia. The features shown were used to train the random forest algorithm used in analysis of dysphagia associations (see text and Appendix S1). CBI‐R, Cambridge Behavioural Inventory–Revised total score; MMSE, Mini‐Mental State Examination score.

Within the nfvPPA group (the only PPA group with a substantial prevalence of dysphagia), patient subgroups with and without dysphagia did not differ significantly in age, sex, years of education, symptom duration, MMSE score, CBI‐R total score, eating behaviours, or neuropsychological profile (Table 1 and Table S1). Those with dysphagia at baseline had a significantly higher prevalence of orofacial apraxia (χ^2^ = 4.36, *p* = 0.037, *V* = 0.552) and parkinsonism (χ^2^ = 6.48, *p* = 0.011, *V* = 0.556). The machine learning algorithm revealed a hierarchy of importance of features predicting dysphagia at baseline in nfvPPA (Figure 1); most important (consistently associated) was the presence of orofacial apraxia, followed by older age, presence of parkinsonism, higher CBI‐R total score, lower MMSE score, decline in table manners, and longer symptom duration. The model correctly predicted eight of nine patients with dysphagia and 11 of 12 without dysphagia (Figure S3), with excellent accuracy (90.5%, 95% CI = 77.9–100), specificity (91.7%, 95% CI = 79.9–100), and sensitivity (88.9%, 95% CI = 75.5–100). In post hoc pairwise comparisons of predictive features for relative importance (Figure S4), the importance score of orofacial apraxia was significantly higher than that of all other features except for age. Older age, parkinsonism, higher CBI‐R total score, and lower MMSE score did not differ significantly from one another in importance score, but all were significantly more important than decline in table manners, increased appetite, sex, or handedness.

### Factors predicting dysphagia during follow‐up (across PPA syndromes)

**FIGURE 2:**
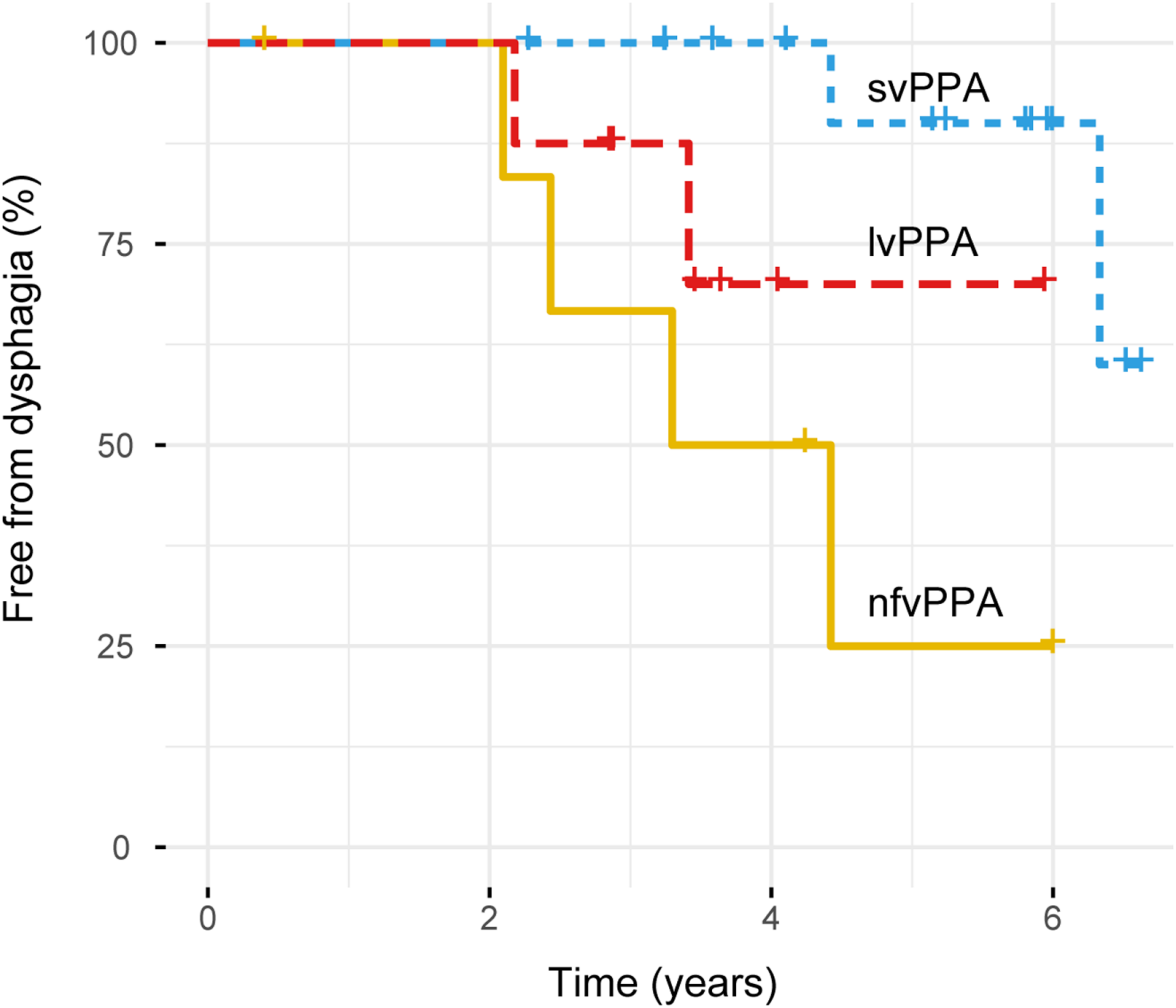
Kaplan–Meier survival analysis for proportions of patients developing dysphagia during follow‐up in each primary progressive aphasia syndromic group: nfvPPA (*n* = 12), svPPA (*n* = 21), and lvPPA (*n* = 13). For patients who did not develop dysphagia, time to dysphagia indicates follow‐up time. Crosses (+) indicate censored cases (patients who were lost to follow‐up). lvPPA, patient group with logopenic variant primary progressive aphasia; nfvPPA, patient group with nonfluent/agrammatic primary progressive aphasia; svPPA, patient group with semantic variant primary progressive aphasia.

Among 46 patients who did not have dysphagia at baseline, 30 were followed clinically (mean [SD] follow‐up interval = 4.19 [1.55] years); mean follow‐up intervals did not differ significantly between syndromic groups (Table 1). Four patients with nfvPPA, two with svPPA, and two with lvPPA developed dysphagia during follow‐up (mean [SD] interval to dysphagia development = 3.6 [1.4] years). The proportion of patients developing dysphagia was significantly higher in the nfvPPA group than the svPPA group (χ^2^ = 9.81, *p* = 0.002) but did not differ significantly between other PPA groups (nfvPPA vs. lvPPA, χ^2^ = 1.15, *p* = 0.28; svPPA vs. lvPPA, χ^2^ = 3.10, *p* = 0.078; Figure 2). The backward multivariate Cox proportional hazard regression model was statistically significant (χ^2^ = 21.84, *p* < 0.001); features significantly associated with development of dysphagia during follow‐up were lower MMSE score (W = 8.05, *p* = 0.005, hazard ratio [HR] = 4.04, 95% CI = 1.34–12.18), presence of orofacial apraxia (W = 7.43, *p* = 0.013, HR = 1.05, 95% CI = 1.00–1.11), and higher CBI‐R total score (W = 4.46, *p* = 0.043, HR = 0.72, 95% CI = 0.58–0.90; further details in Table S2).

### Other clinical associations with dysphagia

Review of clinical records in patients with PPA and dysphagia (at baseline or during follow‐up) extracted information about swallowing function in seven patients with nfvPPA, two with svPPA, and one with lvPPA (Table 2). As anticipated, dysphagia occurred earlier in the course of the illness and was more troublesome in patients with nfvPPA; each of these patients had required at least some dietary or other modifications around mealtimes, and for most, caregivers had adopted compensatory strategies. Although some coughing at meals was evident in the majority, associated weight loss or chest infections were infrequently reported.

### Neuroanatomical associations of dysphagia

**FIGURE 3:**
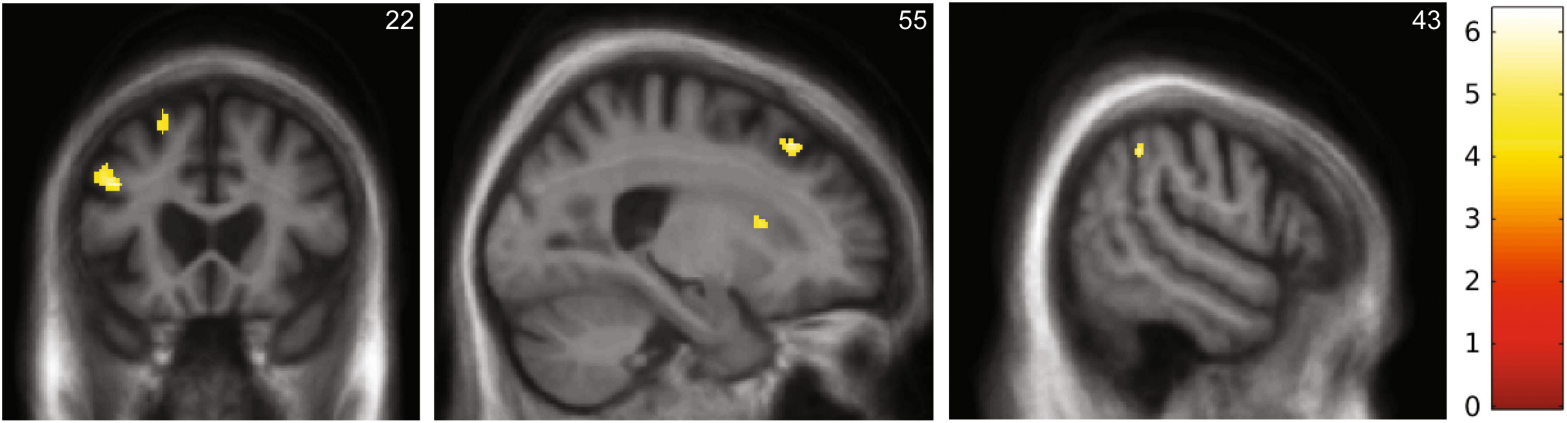
Statistical parametric maps showing regional grey matter differences between subgroups of patients with nonfluent/agrammatic variant primary progressive aphasia who did and did not report dysphagia, based on a voxel‐based morphometric analysis of their brain magnetic resonance (MR) images. For display purposes, maps are thresholded at *p* < 0.001 uncorrected over the whole brain and presented on coronal (left) and sagittal (middle, right) sections of a group (combined patient group) mean T1‐weighted brain MR image; the plane of each section is indicated using Montreal Neurological Institute coordinates, and the left cerebral hemisphere is displayed on the left in the coronal section. The colour bar codes voxelwise *T*‐scores for the atrophy maps. The grey matter associations shown signify more marked regional grey matter loss in patients with dysphagia, and all had local maxima significant at *p* < 0.05, after correction for multiple voxelwise comparisons within the anatomical region of interest (see text and Table 3).

Statistical parametric maps of significant grey matter associations with dysphagia in the nfvPPA group are presented in Figure 3, and local atrophy maxima are summarized in Table 3. Relative to patients without dysphagia, patients with dysphagia had greater regional grey matter atrophy in left middle frontal gyrus, right superior frontal gyrus, right supramarginal gyrus, and right caudate (all significant at *p* < 0.05 after correction for multiple voxelwise comparisons over the prespecified neuroanatomical region of interest). No significant grey matter associations of the reverse contrast (more severe atrophy in patients without dysphagia) were identified at the prescribed corrected significance threshold. Inspection at a relaxed, uncorrected significance threshold *p* < 0.001 over the whole brain revealed no additional regional grey matter associations with dysphagia beyond the prespecified regions of interest.

**TABLE 3:** Neuroanatomical associations of dysphagia in patients with nonfluent/agrammatic variant primary progressive aphasia.

Region	Cluster size, voxels	Peak, mm			*T* score	*p*
		x	y	z		
Left middle frontal gyrus	45	−36	8	37	6.35	0.021
Right superior frontal gyrus	28	24	29	48	6.21	0.024
Right supramarginal gyrus	29	56	−43	45	5.32	0.031
Right caudate	4	8	20	12	4.68	0.033

*Note*: The table summarizes statistically significant neuroanatomical differences between subgroups of patients with nonfluent/agrammatic variant primary progressive aphasia who did and did not report dysphagia, based on a voxel‐based morphometric analysis of their brain magnetic resonance images. All differences reflect more marked regional grey matter loss in patients with dysphagia. All local maxima presented were significant at *p* < 0.05, after correction for multiple voxelwise comparisons within the anatomical region of interest (see text, Figure S1). Coordinates of local maxima are in Montreal Neurological Institute standard space.

## DISCUSSION

Here we have shown that dysphagia is a significant clinical issue in PPA, but strikingly more prevalent in nfvPPA than other syndromes, developing in approximately half of our patients with nfvPPA. Dysphagia in these patients impacted daily life functioning around mealtimes and often led to dietary modifications or other compensatory strategies; however, swallowing difficulties were not always associated with coughing at mealtimes (Table 2), underlining the need for a more searching history to ensure that dysphagia is not overlooked. The most important clinical predictor of dysphagia in patients with nfvPPA (at the time of presentation to a neurologist) was the presence of orofacial apraxia, followed by older age, parkinsonism, more severe behavioural disturbance (higher CBI‐R total score), and/or more severe cognitive impairment (lower MMSE score). Across PPA syndromes, development of dysphagia over the course of the illness was similarly predicted by more severe cognitive impairment (lower MMSE score), the presence of orofacial apraxia, and/or more severe behavioural disturbance (higher CBI‐R total score). Neuroanatomically, development of dysphagia (in the nfvPPA group) was associated with grey matter atrophy in a distributed, bihemispheric prefrontal, inferior parietal, and dorsal striatal network.

The high frequency of dysphagia in nfvPPA in our series is in line with previous evidence in patients with primary progressive apraxia of speech [24], although only a minority of patients with dysphagia here had "pure" apraxia of speech. Our findings further support caregiver survey testimony that dysphagia often manifests relatively early in the course of nfvPPA [3], although only two of our patients fulfilled criteria for "pure" PPA of speech (Table 1). Our findings further endorse caregiver reports that dysphagia becomes more frequent and problematic later in the course of all PPA syndromes, accompanying more severe cognitive and behavioural decline [3, 4]. These associations in PPA corroborate previous work linking deterioration in cognition and behaviour to dysphagia in behavioural variant frontotemporal dementia [25], as well as large longitudinal studies indicating that older age and frailty are nonspecific risk factors for dysphagia [26, 27]. Although our study does not resolve the specific pathophysiological basis for dysphagia in PPA, the profile of predictive features and associations gives clues to a likely mechanism. In particular, the strong association of dysphagia in nfvPPA with orofacial apraxia (and to a lesser degree, parkinsonism) further corroborates previous reports in PPA and primary extrapyramidal syndromes [28, 29, 30, 31, 32, 33, 34]; this evidence suggests that dysphagia in PPA syndromes is likely, at least in part, to reflect disordered motor regulation of complex sequential oropharyngeal and facial actions, exemplified by swallowing, nonverbal orofacial gestures, and the core dysfunction of speech motor output mechanisms that defines nfvPPA.

It is likely a priori that different factors will additionally promote the development of dysphagia in particular PPA syndromes; for example, clinical experience suggests that changes in eating behaviour such as hyperphagia and overfilling of the mouth lead to dysphagia in some patients with PPA (more often, with svPPA [35]), and decline in table manners may be a marker for this. Although the low prevalence of dysphagia in svPPA and lvPPA here precluded more detailed syndrome‐specific analyses, it is noteworthy that this factor was substantially less strongly associated than motor features with dysphagia in nfvPPA.

The underlying mechanism of dysphagia in nfvPPA is further illustrated by the profile of neuroanatomical correlates in the VBM analysis. After adjusting for potentially confounding factors of older age and longer disease duration, development of dysphagia in our nfvPPA group was associated with greater atrophy in a corticosubcortical network that has previously been implicated in the normal control of swallowing and its dysregulation in neurological disorders, as well as the development of speech apraxia in PPA. Middle frontal gyrus participates in regulating both reflexive and volitional swallowing in the healthy brain [11, 12]; it is a key focus of neurodegenerative pathology in nfvPPA [36], and atrophy involving this region has also been correlated with orofacial apraxia in other PPA cohorts [21, 31]. The additional involvement of right‐lateralized areas here aligns with previous work showing that right hemisphere stroke is a strong predictor of dysphagia [37, 38]. Superior frontal gyrus has been implicated in the initiation of swallowing in Parkinson disease and after stroke [39, 40]; in concert with more posterior parietal areas including supramarginal gyrus and basal ganglia structures including the caudate nucleus, prefrontal cortices link swallowing motor programmes with spatiotemporal movement planning and execution of coordinated muscle activity, guided by sensory feedback [12].

This study has several limitations that should direct future work. Our characterization of dysphagia was based largely on caregiver reports and clinical records; a full understanding of dysphagia in PPA will require prospective, objective evaluation of swallowing function in larger PPA cohorts, engaging a speech and language therapist with grading of dysphagia severity. This should be coupled with a more systematic and comprehensive assessment of pertinent accompanying deficits (such as orofacial apraxia) and daily life impact. Particular motor features within the parkinsonian PSP‐CBS spectrum (for example, "axial" features such as gait and postural abnormalities) could be assessed as predictors of dysphagia, with a view to the underlying mechanism. Functional neuroimaging might delineate and discriminate the neural mechanisms underpinning automatic and volitional swallowing and the different phases of deglutition. Our findings should be replicated in larger PPA cohorts, with tracking of how the symptom emerges and evolves longitudinally in individual patients, alongside other neurological features such as parkinsonism; it should be noted that we did not follow patients into the final stages of their illness, when issues around nutrition, aspiration events, and the question of assisted feeding are likely to become more salient. We need more information about how dysphagia manifests in svPPA and lvPPA, and in different subtypes of nfvPPA with predominant speech apraxia versus agrammatism [2].

This study foreshadows a need for greater awareness of dysphagia among clinicians who care for patients with PPA. Given the serious (even potentially life‐threatening) consequences of dysphagia if undetected or inadequately managed, care pathways for PPA should foreground optimization of swallowing function as a matter of urgency. An essential component of this will be early and judicious referral to a speech and language therapist, who can assist with both the assessment of swallowing and interventions such as the prescription of thickening agents, dietary modifications, behavioural strategies, and discussion of alternative feeding options. Currently, however, access to speech and language therapy by people living with PPA is limited, with considerable geographical variation in the United Kingdom [41].

In conclusion, our findings call attention to a key nonlinguistic feature of PPA that potentially impacts everyday functioning and prognosis, and illuminates underlying pathophysiological mechanisms. Clinicians should be alert to sentinel symptoms such as orofacial apraxia that signal a need for assessment of swallowing function in PPA, particularly in nfvPPA and in the setting of more severe disease. The importance of dysphagia in these syndromes warrants further study in larger prospective, multicentre cohorts and signposting in management guidelines and care pathways for PPA, acknowledging the role of speech and language therapy.

## AUTHOR CONTRIBUTIONS

**Salvatore Mazzeo:** Conceptualization; investigation; writing – original draft; methodology; validation; formal analysis; data curation. **Eoin Mulroy:** Conceptualization; investigation; methodology; visualization; writing – review and editing; data curation; resources. **Jessica Jiang:** Writing – review and editing; resources; visualization; methodology; data curation. **Michael Lassi:** Methodology; visualization; writing – review and editing. **Jeremy C. S. Johnson:** Resources; data curation; writing – review and editing; visualization. **Chris J. D. Hardy:** Writing – review and editing; methodology; visualization; supervision; resources; data curation. **Jonathan D. Rohrer:** Resources; visualization; writing – review and editing. **Jason D. Warren:** Conceptualization; investigation; funding acquisition; writing – review and editing; visualization; methodology; project administration; supervision; resources; data curation. **Anna Volkmer:** Data curation; supervision; resources; project administration; visualization; methodology; writing – review and editing; funding acquisition; investigation; conceptualization.

## FUNDING INFORMATION

This work was supported by the Alzheimer's Society, Alzheimer's Research UK, the UK Dementia Research Institute at UCL, and the National Institute for Health Research (NIHR) University College London Hospitals Biomedical Research Centre. J.J. was supported by a Frontotemporal Dementia Research Studentship in Memory of David Blechner (funded through The National Brain Appeal). J.C.S.J. was supported by an Association of British Neurologists Clinical Research Training Fellowship. C.J.D.H. is supported by an NIHR (Invention for Innovation [NIHR204280]) grant and received a Wellcome Institutional Strategic Support Fund Award (204841/Z/16/Z). J.D.R. has been supported by a Miriam Marks Brain Research UK Senior Fellowship, a Medical Research Council Clinician Scientist Fellowship (MR/M008525/1), and the NIHR Rare Disease Translational Research Collaboration (BRC149/NS/MH). J.D.W. is supported by the Alzheimer's Society, Alzheimer's Research UK, the Royal National Institute for Deaf People (Discovery Grant G105_WARREN), and the NIHR University College London Hospitals Biomedical Research Centre. A.V. is supported by an NIHR Advanced Fellowship NIHR302240. This research was funded in part by UKRI and Wellcome Trust (Grant 204841/Z/16/Z). For the purpose of Open Access, the authors have applied a Creative Commons Attribution (CC BY) public copyright licence to any author‐accepted manuscript version arising from this submission. The views expressed are those of the authors and not necessarily those of the NIHR or the Department of Health and Social Care.

## CONFLICT OF INTEREST STATEMENT

The authors report no competing interests.

## Supporting information


Appendix S1.

## Data Availability

Anonymized data that support the findings of this study will be shared on request from any qualified investigator.
