# Supplementary material for: Dysphagia in primary progressive aphasia: Clinical predictors and neuroanatomical basis
Source: Eur J Neurol. 2024 Jun 21;31(9):e16370. doi: 10.1111/ene.16370 (PMC11295169; doi:10.1111/ene.16370)

**Supplementary Material. Dysphagia in primary progressive aphasia: Clinical predictors and neuroanatomical basis, by S Mazzeo et al**

**Details of brain image acquisition and pre-processing**

Volumetric brain MRI scans were acquired from patients on a 3T MAGNETOM Prisma scanner (Siemens Healthcare, Erlangen, Germany) using a 64-channel head-and-neck receiver array coil and a T1-weighted sagittal 3D magnetization-prepared rapid gradient-echo sequence (echo time = 2.93 ms, inversion time = 850 ms, repetition time = 2000 ms), with matrix size 256 × 256 × 208 and voxel dimensions 1.1×1.1×1.1 mm and overall scan acquisition duration 306 seconds.

Brain images were first pre-processed and normalised to Montreal Neurological Institute (MNI) space with isotropic voxel size 1.5 mm using SPM12 software (http://www.fil.ion.ucl.ac.uk/spm/software/spm12/) and the Diffeomorphic Anatomical Registration Through Exponentiated Lie Algebra (DARTEL) toolbox with default parameters in MATLAB R2014b (MathWorks, Natick, MA, USA), following a previously described protocol [1,2]. To control for individual differences in total (pre-morbid) brain size, total intracranial volume was calculated for each participant by summing white matter, grey matter and CSF volumes post-segmentation [3]. An explicit brain mask was created using an automatic mask-creation strategy designed previously [4]. A study-specific mean brain template image upon which to overlay statistical parametric maps was created by warping all patients’ native-space whole-brain images to the final DARTEL template and using the ImCalc function to generate an average of these images

**Details of the machine learning model**

Missing values (where these accounted for <20% of the total) were replaced with the median of the respective column. All continuous variables were standardised by subtracting the group mean value and dividing by the standard deviation. To address the issue of class imbalance in the dataset, the Synthetic Minority Over-sampling Technique was applied [5]. A Random Forest classifier (configured with 500 decision trees and a random state of 1, split criterion [Gini], minimum number of samples for splitting (2), bootstrap (True)) was employed for the classification task. To evaluate the performance of the classification model, a Leave-One-Out cross-validation strategy was employed to generate the training and test sets for each fold; for each fold, one sample was left out as the test set, and the remaining samples were used for training.

1 Hardy CJD, Agustus JL, Marshall CR, *et al.* Behavioural and neuroanatomical correlates of auditory speech analysis in primary progressive aphasias. *Alzheimers Res Ther*. 2017;9:53.

2 Hardy CJD, Agustus JL, Marshall CR, *et al.* Functional neuroanatomy of speech signal decoding in primary progressive aphasias. *Neurobiol Aging*. 2017;56:190–201.

3 Malone IB, Leung KK, Clegg S, *et al.* Accurate automatic estimation of total intracranial volume: a nuisance variable with less nuisance. *Neuroimage*. 2015;104:366–72.

4 Ridgway GR, Omar R, Ourselin S, *et al.* Issues with threshold masking in voxel-based morphometry of atrophied brains. *Neuroimage*. 2009;44:99–111.

5 Chawla NV, Bowyer KW, Hall LO, *et al.* SMOTE: Synthetic Minority Over-sampling Technique. *jair*. 2002;16:321–57.

**Table S1. Neuropsychological characteristics of patient groups**

| **Cognitive domain** | **nfvPPA** | | | **svPPA** | **lvPPA** |
| --- | --- | --- | --- | --- | --- |
|  | **Combined** | **Dysphagia at baseline** | **No dysphagia at baseline** |  |  |
| ***Executive*** |  |  |  |  |  |
| WASI: Matrices (/32) | 13.5 (8.4)^a^ | 15.4 (6.2) | 12.2 (9.79) | 22.9 (8.3)^a,b^ | 13.5 (5.9)^b^ |
| Trail Making Test A  (max 150s) | **75.5 (46.3)** | **98.0 (57.0)** | **56.3 (25.5)** | **53.8 (31.1)** | **90.5 (36.3)** |
| Trail Making Test B  (max 300s) | **144.0 (103.0)** | **113 (64.7)** | **164.0 (123.0)** | **137 (91.0)** | **128.0 (88.8)** |
| Phonological fluency (60s) | **6.1 (4.8)** | **7.7 (8.1)** | **5.4 (2.6)** | **9.33 (5.2)** | **5.00 (4.2)** |
| Category fluency (60s) | **10.1 (5.9)** | **9.7 (7.3)** | **10.2 (5.7)** | **7.19 (4.9)** | **6.73 (5.8)** |
| ***Language skills*** |  |  |  |  |  |
| Graded Naming Test (/30) | **13.5 (7.5)^i^** | **14.1 (7.8)** | **12.9 (7.5)** | **2.3 (4.9)^i^** | **7.2 (7.5)** |
| BPVS (/150) | **125.7 (35.4)^g^** | **139.0 (11.5)** | 115.0 (44.5) | **87.9 (50.6)^g,h^** | 129.2 (29.6)^h^ |
| Sentence construction (written sentences) (/25) | **16.7 (9.1)** | **16.0 (10.7)** | **17.2 (8.7)** | **20.5 (5.8)** | **17.9 (3.4)** |
| Sentence comprehension  (PALPA-55) (/24) | **17.5 (5.5)^o^** | **20.0 (3.3)** | **15.9 (6.2)** | **22.1 (3.2)^o,p^** | **15.8 (5.4)^p^** |
| Word repetition (/45) | **35.0 (11.6)^n^** | **40.0 (7.8)** | **32.5 (12.8)** | 43.3 (3.1)^n^ | **37.5 (9.4)** |
| Graded difficulty sentence repetition (/10) | **4.13 (2.9)^q^** | **5.4 (2.1)** | **3.5 (3.2)** | **8.06 (1.8)^q,r^** | **4.17 (1.8)^r^** |
| NART (/50) | 21.9 (12.9) | 26.8 (11.9) | 19.5 (13.4) | 21.1 (14.7) | 22.2 (12.0) |
| Spelling (/30) | 17.7 (7.3) | 19.0 (9.3) | 16.9 (6.5) | 13.9 (7.1) | 13.3 (5.6) |
| ***Working memory*** |  |  |  |  |  |
| Digit span (forward) (/12) | 4.9 (2.5)^c^ | 5.4 (2.5) | 4.4 (2.5) | 8.7 (2.3)^c,d^ | **3.3 (2.0)^d^** |
| Digit span (reverse) (/12) | 3.6 (1.5)^e^ | 4.0 (1.1) | **2.9 (1.7)** | 6.1 (2.0)^e,f^ | **2.9 (1.3)^f^** |
| ***Episodic memory*** |  |  |  |  |  |
| Camden PAL (/24) | **14.5 (6.3)** | **11.4 (8.2)** | 17.2 (2.7) | **5.9 (7.1)** | **7.0 (7.0)** |
| RMT words (/50) | 41.2 (7.1) | 40.0 (6.1) | 41.4 (8.26) | **36.5 (7.3)** | **36.9 (9.7)** |
| RMT faces (/50) | **34.9 (7.4)** | **34.3 (5.0)** | **35.5 (8.8)** | **34.0 (7.2)** | **33.1 (7.0)** |
| ***Other skills*** |  |  |  |  |  |
| Graded Difficulty Arithmetic (/24) | 5.9 (5.6)^j^ | 9.3 (6.7) | **3.7 (3.5)** | 11.4 (7.3)^j,k^ | **2.3 (2.1)^k^** |
| VOSP: Object Decision (/20) | 15.0 (3.6) | **13.7 (3.9)** | 15.8 (3.7) | 17.1 (3.31) | 15.4 (2.8) |

The table presents mean (standard deviation) values unless otherwise indicated; maximum scores are also indicated alongside the corresponding tests (in parentheses). Bold indicates values falling below the 10^th^ percentile for healthy control norms. For all comparisons between patient groups, the statistical significance criterion for significant group differences was p < 0.05. ^a^ p < 0.001, d = 1.19; ^b^ p= 0.004, d = 1.20; ^c^ p < 0.001, d = 1.67; ^d^ p = 0.001, d = 2.35; ^e^ p < 0.001, d = 1.62; ^f^ p < 0.001, d = 1.88; ^g^ p = 0.020, d = 0.091, ^h^ p = 0.024, d = 1.00; ^i^ p < 0.001, d = 1.69; ^j^ p = 0.034, d = 0.91; ^k^ p = 0.002, d = 1.50; ^l^ p < 0.001, d = 1.83; ^m^ p < 0.001, d = 1.35; ^n^ p = 0.018, d = 0.98; ^o^ p = 0.0.26, d = 0.96; ^p^ p = 0.004, d = 1.33; ^q^ p < 0.001, d = 1.71; ^r^ p < 0.001, d = 1.70; ^s^ p < 0.001, d = 1.35; ^t^ p < 0.001, d = 1.56; ^u^ p = 0.023, d = 0.95; ^v^ p < 0.001, d = 1.65; ^w^ p = 0.026, d = 1.39. BPVS, British Picture Vocabulary Scale; lvPPA, patient group with logopenic variant primary progressive aphasia; NART, National Adult Reading Test; nfvPPA, patient group with nonfluent/agrammatic primary progressive aphasia; PAL, Paired Associate Learning; PALPA, Psycholinguistic Assessments of Language Processing in Aphasia (subtest 55); RMT, Recognition Memory Test; svPPA, patient group with semantic variant primary progressive aphasia; VOSP, Visual Object and Space Perception battery; WASI, Wechsler Abbreviated Scale of Intelligence.

**Table S2. Significant predictors of dysphagia development during follow-up in the combined primary progressive aphasia cohort**

| **Association** | **B** | **Wald** | **p** | **HR** | **95% C.I.** | |
| --- | --- | --- | --- | --- | --- | --- |
|  |  |  |  |  | **lower** | **upper** |
| MMSE | -0.327 | 8.050 | 0.005 | 0.721 | 0.576 | 0.904 |
| Orofacial apraxia | 0.049 | 7.429 | 0.013 | 4.044 | 1.343 | 12.178 |
| CBI-R total score | 0.655 | 4.456 | 0.043 | 1.053 | 1.002 | 1.108 |

This table summarises the results of a backward Cox’s proportional hazard regression analysis to identify factors predicting development of dysphagia during follow-up in those patients (across the PPA cohort) who did not have dysphagia at the baseline research assessment. The table shows regression coefficients (B), Wald coefficients (Wald), p-values (p), hazard ratios (HR) and 95% confidence intervals (95% C.I.) for covariates included in the regression model. The regression model overall had χ^2^ value 21.8 and significance level p < 0.001. The statistical significance threshold was set at p < 0.05. MMSE, Mini-Mental State Examination score; CBI-R, Cambridge Behavioural Inventory (Revised).

**Figure S1.** Representative sections of neuroanatomical regions in the left and right cerebral hemispheres that were used for multiple voxel-wise comparisons correction in region-of-interest analyses (see text). Regions are rendered on coronal (upper left), sagittal (upper right) and axial (bottom) sections of the mean normalised brain template for the patient cohort; MNI coordinates of the plane of each section are shown. The neuroanatomical regions comprise bilateral frontal pole (red), superior frontal gyri (dark blue), middle frontal gyri (light green), inferior frontal gyri pars opercularis (yellow), inferior frontal gyri pars triangularis (purple), precentral gyri (light blue), supramarginal gyri (white), basal ganglia (dark green).

**
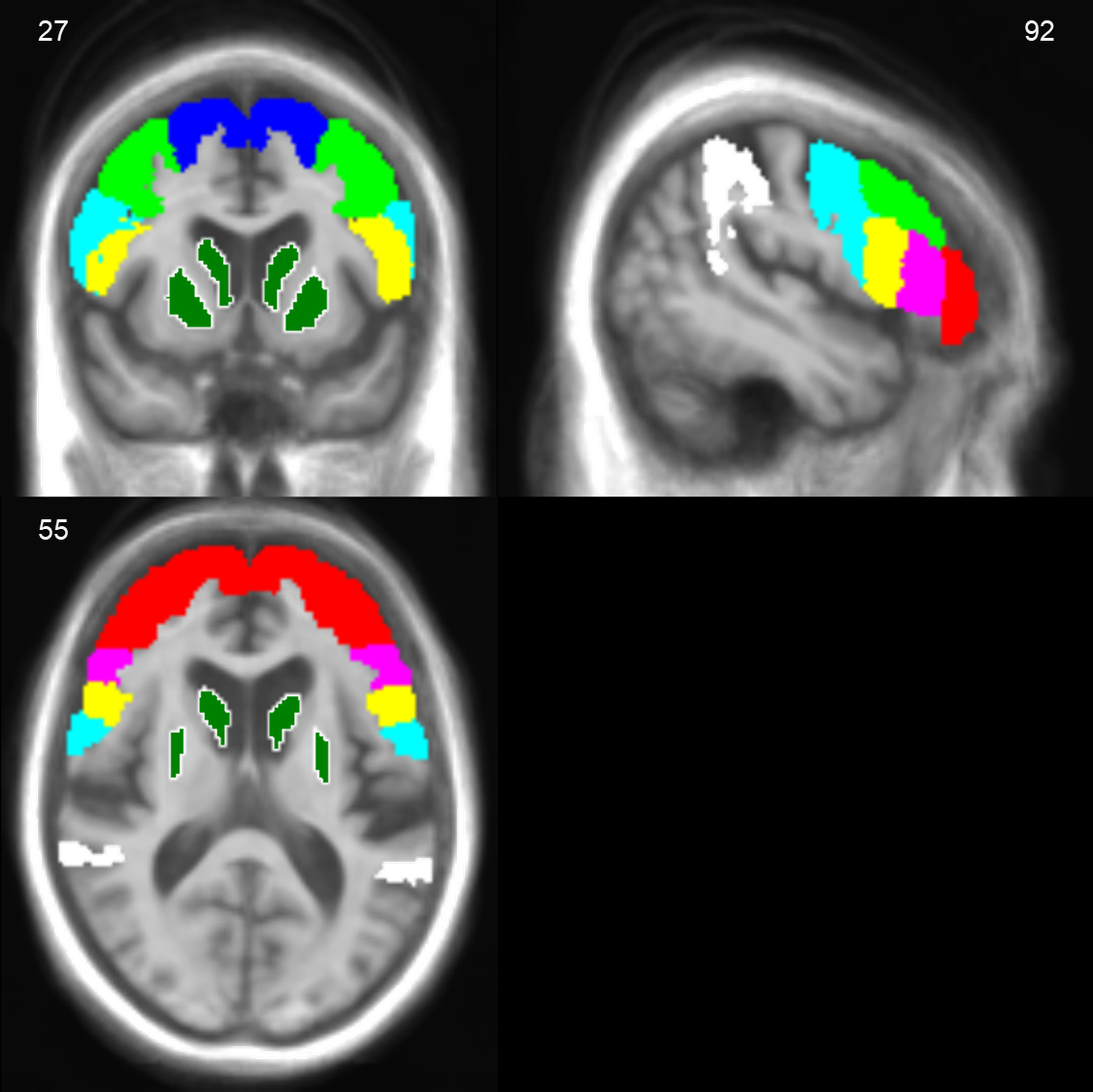
**

**Figure S2.** Diagram describing the sample with frequencies of patients with dysphagia at baseline and during follow-up. lvPPA, logopenic variant primary progressive aphasia; nfvPPA, nonfluent/agrammatic variant primary progressive aphasia; svPPA, semantic variant primary progressive aphasia.

**
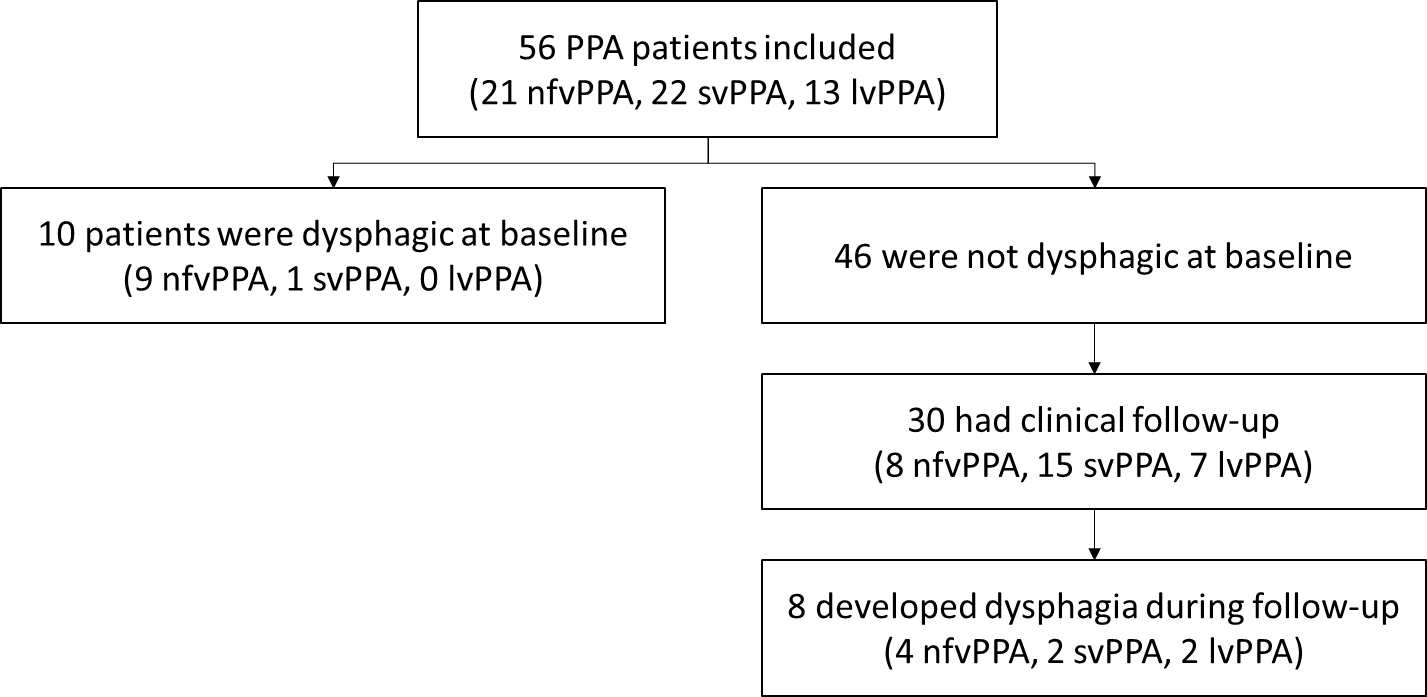
**

**Figure S3.** Confusion matrix output of the machine learning model for dysphagia at presentation in patients with nfvPPA. The values in the matrix represent the number of patients in each category, colour coded according to the scale on the right. "True label" indicates the clinical classification of patients as "dysphagic" or "not dysphagic," as described in Methods; "Predicted label" indicates the classification generated by the machine learning model (see main text).


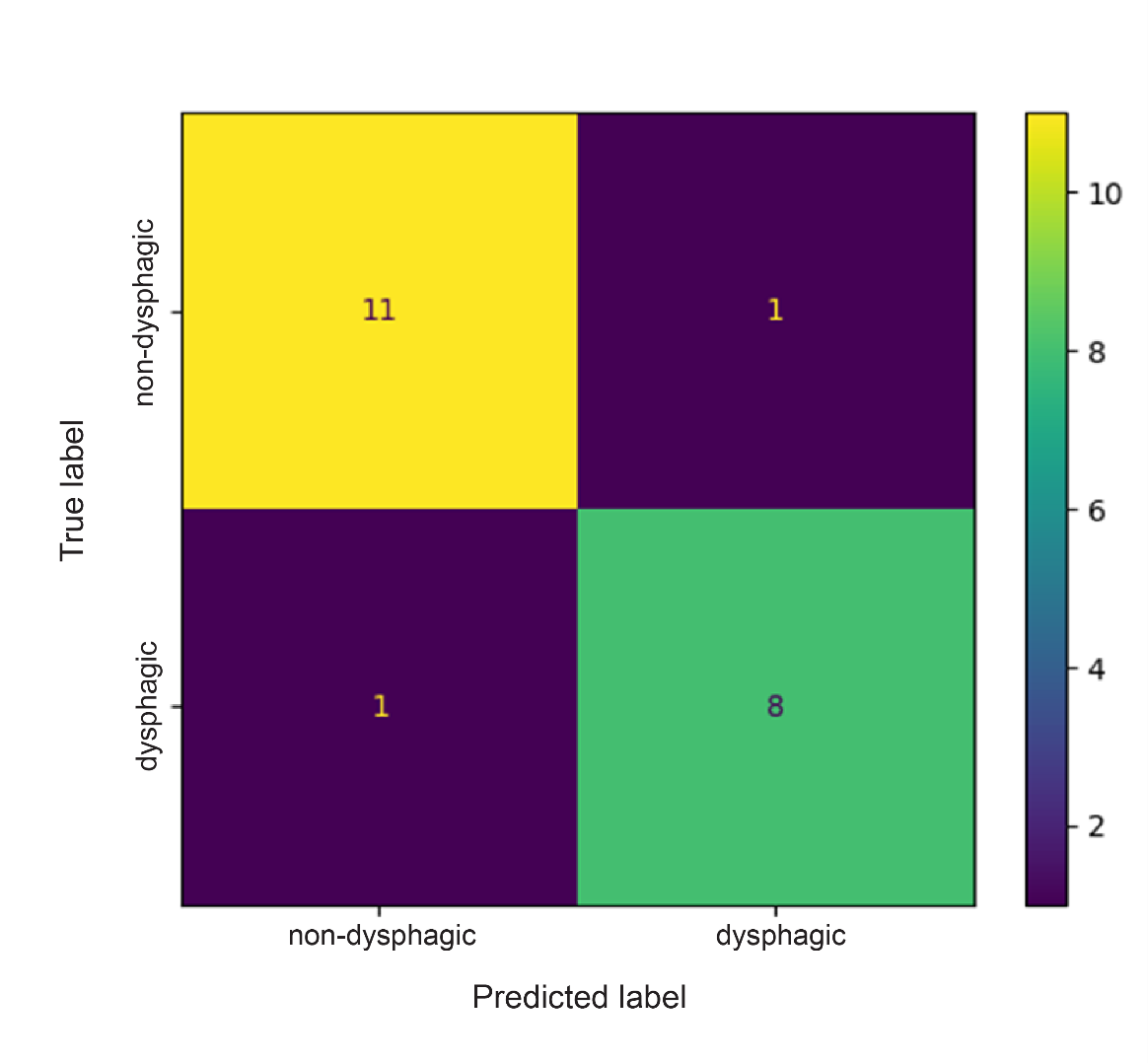


**Figure S4.** Bonferroni-corrected pairwise comparisons of Importance of features predicting dysphagia at presentation in nfvPPA patients, from the machine learning analysis. p-values for feature comparisons are shown; an upward arrow (↑) indicates that the feature listed in the row is more important than the feature listed in the column, and number of arrows indicates the level of significance after Bonferroni correction (↑: p < 0.05, ↑↑: p < 0.01, ↑↑↑: p < 0.001). CBI-R, Cambridge Behavioural Inventory (Revised) total score; MMSE, Mini-Mental State Examination score.


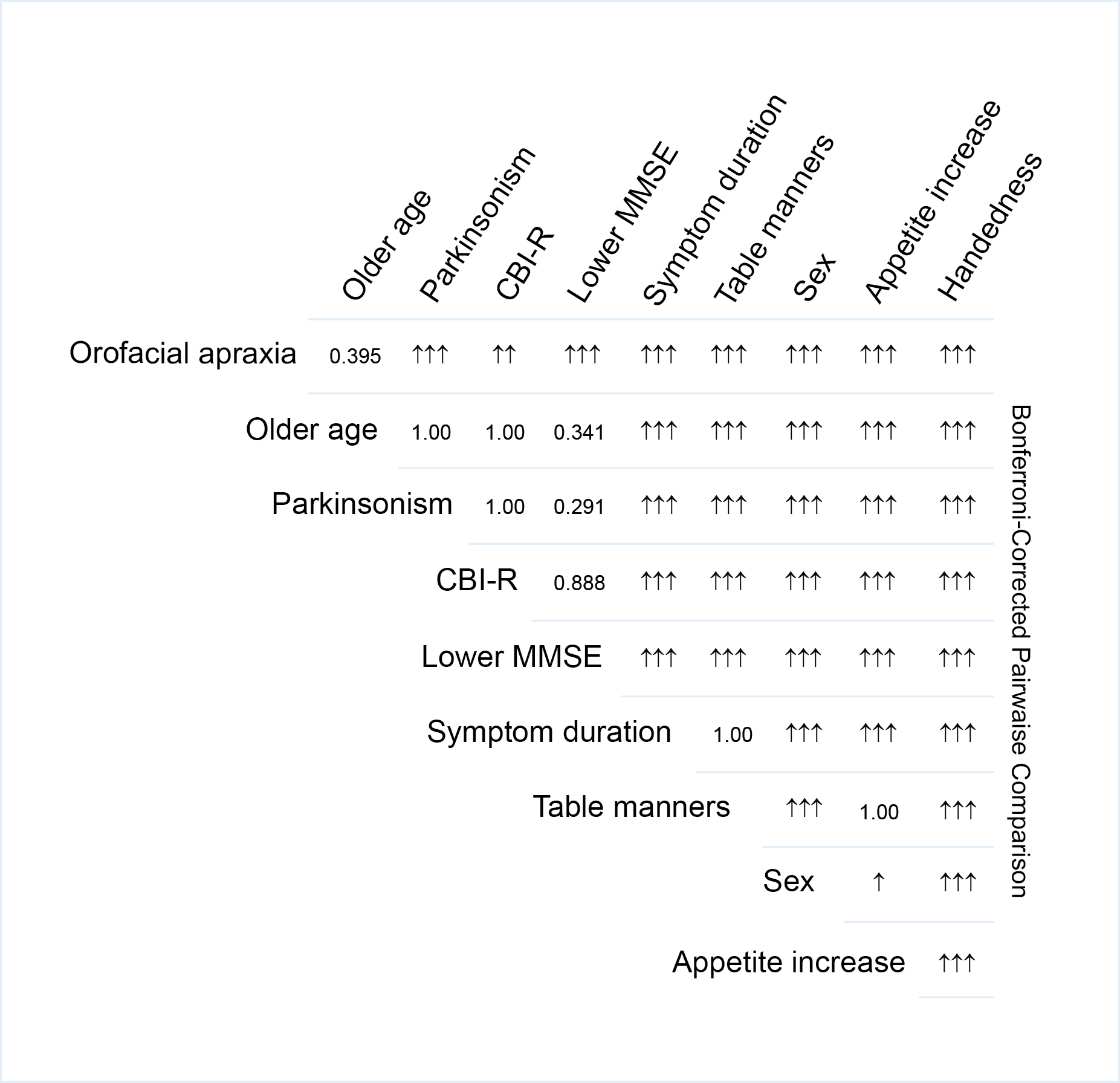

Supplement: Supplementary file 1 — Appendix S1. [file ENE-31-e16370-s001.docx]
